# Different Susceptibilities of Human Melanoma Cell Lines to G2/M Blockage and Cell Death Activation in Response to the Estrogen Receptor β agonist LY500307

**DOI:** 10.7150/jca.65425

**Published:** 2022-03-06

**Authors:** Giada Pontecorvi, Maria Bellenghi, Sabrina Tait, Valentina Tirelli, Paola Matarrese, Gianfranco Mattia, Alessandra Carè, Rossella Puglisi

**Affiliations:** 1 Center for Gender-Specific Medicine, Istituto Superiore di Sanità, Rome, Italy; 2 Core facilities, Istituto Superiore di Sanità, Rome, Italy

**Keywords:** melanoma, LY500307, Estrogen Receptors, cell cycle, apoptosis.

## Abstract

**Background:** Gender differences in melanoma incidence, metastasis formation and disease progression are increasingly evident in epidemiological studies, with women showing significantly better survival than men. Among factors possibly underlying the disparities, sex hormones seem to play a key role. Nonetheless, functional mechanisms are still unclear, except for the antitumor ability of Estrogen Receptor (ER) β, whose expression determination has often been suggested for melanoma prognosis. In this study, we aimed at evaluating the molecular mechanisms and functional effects associated with ERβ signaling by using its agonist LY500307.

**Methods:** We evaluated the antitumor effect of the specific synthetic ERβ agonist LY500307 on some human melanoma cell lines, selected for different genetic background, expression levels of ERs and tumor progression. The expression of α and β estrogen receptors was investigated taking advantage of The Cancer Genome Atlas database and confirmed on some selected melanoma cell lines. The biological effects of LY500307 were determined *in vitro* looking at melanoma cell proliferation, cell cycle profiles and migration demonstrating by western blot the involvement of some pathway specific markers. The LY500307-dependent induction of cell death was also analyzed by flow cytometry and western blot analysis of caspase 3 and poly adenosine diphosphate-ribose polymerase (PARP).

**Results:** A significant decrease in the expression of both ERs, even more pronounced for ERα, has been found in patients with metastatic NRAS mutation. Treatment with LY500307 significantly reduced the proliferation of melanoma cells showing a cell cycle arrest at the G2/M boundary phase and promoting apoptosis with different sensitivities associated with disease stage and mutation*.* Indeed, the ERβ agonist affects melanoma migration, inducing a reversion of the epithelial-mesenchymal transition, more evident in a low aggressive primary melanoma cell line.

**Conclusion:** These results demonstrate the capability of LY500307 to reduce melanoma malignancy, counteracting cell viability and dissemination, overall suggesting a possible future use of LY500307 in personalized combined therapy.

## Introduction

Cutaneous melanoma is the most aggressive and deadly type of skin cancer, mainly due to its high heterogeneity and remarkable propensity for metastatic spreading. Epidemiological data show sex divergences in both melanoma incidence and mortality rate, being men the most affected 1. Searching for factors responsible for gender differences, a pivotal role of sex hormones has been suggested, indicating melanoma as a hormone-related cancer. Obviously, the involvement of other mechanisms cannot be excluded, leaving still unresolved the actual relevance of the hormonal influence on melanoma onset and progression. Sex influence on melanoma incidence and progression depends on both the hormonal concentration in the bloodstream and the expression of its receptors. Indeed, 17β-estradiol, the main active circulating form of estrogen, plays an important protective role by strengthening the immune system 2. Unfortunately, its therapeutic application is severely limited by enormous risks of developing other types of cancer, especially in women. Furthermore, as estrogen ability to bind with a similar affinity both estrogenic receptor α (ERα) and β (ERβ), pro- and antiproliferative respectively, it can evoke opposite effects in melanoma 3. Therefore, determining the relative expression of each ER, more than their absolute amounts, could better predict estrogenic stimulus outcomes in a given tissue.

Looking at ERα, its presence or absence in both primary and metastatic melanoma specimens remains an unresolved issue 4. However, the presence of ERα mRNA and the increase of epigenetic control of its promoter with disease progression have been demonstrated 5, 6. Conversely, several studies conducted on human melanoma tissues agree on ERβ decreased expression compared to healthy perilesional skin and, more generally, on the presence of an inverse correlation between ERβ levels and Breslow thickness 7, 8. According to the survival advantage of female melanoma patients, men show significantly lower levels of ERβ in both melanoma and healthy tissues 7. As widely known, melanoma presents a high percentage of genetic mutations. Although men accumulate a higher number of missense mutations (ratio Men to Women 1.85), their presence in metastatic melanoma results beneficial only for the overall survival in women, once again supporting the relevance of the functional pressure of the more efficient female immune system 9. Conversely, the most common mutations involving B-Raf Proto-Oncogene (BRAF) and NRAS Proto-Oncogene (NRAS) seem to occur regardless of sex 10. These “driver mutations” lead to constitutive activation of mutant signaling proteins that induce pathways supporting tumor onset 11. Several efforts have been made to develop promising melanoma therapies, such as the combined target therapy against BRAF and Mitogen-Activated protein kinase (MEK) 12-14. Regarding NRAS mutations, generally linked to poorer overall survival, targeted therapies based on MEK inhibition as monotherapy or in combination with Mitogen Activated Protein Kinase (MAPK), Phosphoinositide 3-kinase (PI3K) or Cyclin Dependent Kinase (CDK) 4/6 inhibitors are under development although with disappointing results 15. To overcome the specificity of each mutation, important results were obtained through different types of immunotherapy, like the immune checkpoint inhibitors (ICIs), really representing an improvement in anticancer strategies 16, 17. Despite this progression in immuno- and target therapies, looking for the molecular mechanisms involved in different pathways of estrogen-dependent signaling in melanoma could help to understand the observed sex differences in disease severity, adverse events and outcomes, eventually opening an avenue towards a more personalized medicine. An important question to address concerns the existence of any relationship between estrogen receptor expression and oncogenic mutational status during disease progression. In this paper, we conducted a bioinformatics analysis in the Skin Cutaneous Melanoma (SKCM) patient database (The Cancer Genome Atlas, TCGA) to investigate both ERα and β expressions in the total melanoma patient population and in BRAF and NRAS mutated subgroups, focusing on their modulation from primary to metastatic stage.

The unquestionable awareness of ERβ antitumor role led to the idea that any ligand capable of increasing ERβ expression or activity could be of great therapeutic utility, not only against melanoma. However, in the past years, many researchers experimented with different types of hormone agonists/antagonists or metabolites on melanoma, without reaching conclusive results 18. Among them, the synthetic non-steroidal selective ERβ agonist LY500307 recently showed promising results, suppressing lung metastases in a mouse model of wild-type melanoma cell line, thanks to innate immunity increase 19. Accordingly, LY500307 affects tumor growth in human glioblastoma and triple negative breast cancer 20, 21, both hormone sensitive tumors. Therefore, the exploitation of ERβ anticancer potential could represent a fruitful choice to counteract human melanoma, also in relation to melanoma mutational state.

Here, we unveil the possible molecular mechanism associated with ERβ signaling by using its agonist LY500307 on some human melanoma cell lines, characterized by different levels of ERs expression, genetic background and tumor progression. Interestingly, we demonstrated a selective effect of LY500307 in melanoma cell lines in terms of cell cycle blockage, apoptosis induction and partial EMT reversion, with particular reference to those expressing ERβ in NRAS-mutated genetic background.

## Methods

### Cell lines and culture conditions

Human melanoma cell lines used in the current study were previously described 22-24 (Table S1). Melanoma cell lines were periodically authenticated by standard short tandem repeat (STR)-based genotyping and the experimental analyses were always performed on controlled samples. Human fibroblast cell line (HF) used as normal control was kindly provided by the laboratory of Biotechnologies of the Experimental Medicine Department (“Sapienza”, University of Rome, Italy) with patient written informed consent and ethical statement 25. All the cells were tested for mycoplasma contamination with MycoAlert kit (LT07-418, Lonza) before use in experiments. Human melanoma cell lines were seeded in 6-well culture dishes in Dulbecco's modified Eagle's medium (DMEM, 61965-026) 2% fetal bovine serum (FBS, 10270-106) v/v and maintained in a humidified incubator (5% CO_2_, 37°C). After three days, the cells were treated with LY500307 (2, 4, 8 μM) (Erteberel, Cayman chemical, 22130), Pyrazolo[1,5-α] pyrimidines (PHTPP, 2.5 and 5 μM) (Selleckchem, 2662), Methyl-Piperidino-Pyrazole (MPP) dihydrochloride (5μM) (Tocris, 1991) or their vehicle (ethanol or DMSO) for different times (5, 24h) for cell cycle analyses and apoptosis assays. LY500307 was also used on normal human fibroblast and melanoma cell lines for proliferation/viability index evaluation (colorimetric assay XTT-based Roche Molecular Biochemicals, Mannheim, Germany).

### Flow Cytometry

#### Cell cycle analysis

Melanoma treated cells were trypsinized and harvested in phosphate buffer saline (PBS), followed by fixation in ice-cold 70% ethanol overnight at 4°C. About 3x10^5^ cells were stained with a mixture of Propidium Iodide (50µg/mL, Sigma-Aldrich, P4864) and RNAse A (200µg/mL, Unimed, 501500) for 30 minutes at room temperature in the dark. The PI-stained cells were analyzed by flow cytometry (FCM) (GalliosTM, BD, San Jose, CA).

#### Annexin V apoptosis assay

Melanoma treated cells were trypsinized and washed twice in PBS1X. A proper number of cells (3-5x10^5^) were resuspended in a mixture of Annexin binding buffer 1X (BD Biosciences, 556454), Annexin V647 (Life Technologies, A23204) and PI (Sigma Aldrich, P4864) according to manufacturer's instructions. Cells were gently vortexed and incubated for 15 minutes at room temperature in the dark. The stained cells were analysed by using flow cytometry (GalliosTM, BD, San Jose, CA). This assay enables identification of both early (AV positive/PI negative), late (AV positive/PI positive) and necrotic (PI positive) cells.

#### FCM analysis of extracellular E-cadherin

Me1402/R melanoma cells were treated with LY500307 for 24h, trypsinized and washed twice in PBS1X. About 5x10^5^ unfixed cells were stained on ice with primary antibody anti-E-cadherin recognizing the extracellular epitope and, subsequently, with the specific secondary antibody. Finally, cells were incubated with LIVE/DEAD™ Viability/Cytotoxicity Kit (molecular probes by Life Technologies, L34962) and analyzed by CytoFLEX LX Flow Cytometer (UV-Violet-Blue-Yellow Green-Red (U-V-B-Y-R) Series, Beckman Coulter).

#### Migration/Scratch assays

Scratch wound assays were performed to measure migration and spreading capabilities of melanoma cells *in vitro*. The cells were seeded in 12-well culture plates and cultured in DMEM 10% FBS v/v to nearly confluent cell monolayer. After that, a linear wound was generated in the cell monolayer (70-80% of confluence) with a sterile p200 pipette tip. Any debris remaining in suspension were removed by washing cells once with PBS and then simple DMEM was replaced with DMEM 2%FBS with LY500307. Three representative images from the scratched area were captured (Bulldog Bio JuLi Smart fluorescent cell analyzer) to estimate the relative migration cells at different time points (t0 and 24h). Data were analyzed quantitatively by using ImageJ (NIH) software, considering for each image the distances between one side of the scratch and the other.

### Western Blot and immunofluorescence analysis

Western blotting was performed according to standard procedures. Total cells lysates were prepared by using NP40 cell lysis buffer, quantified by Bradford method and separated by the precast NuPAGE polyacrylamide gel system (Life Technologies Carlsbad, CA, USA). The expression levels were quantified using the AlphaView software (ProteinSimple San Josè, CA, USA).

Immunofluorescence analysis was performed according to standard procedures. Semi-confluent cells were grown and treated with LY500307 in 8-well chamber slides (Nalgene Nunc) and subsequently fixed in 4% (w/v) paraformaldehyde (PFA, Sigma-Aldrich), permeabilized and saturated at room temperature. Incubations with primary and specific fluorophore-conjugated secondary antibodies (Alexa Fluor, Molecular Probes Eugene, OR, USA) were done in a humidified chamber at room temperature. Finally, cells were incubated with Hoechst 33342 to stain DNA and slides were mounted with ProLong without DAPI (Invitrogen, P36930). Cellular staining was analyzed by Olympus FV1000 laser-scanning confocal microscopy (Olympus, Tokyo, Japan).

### List of Utilized Antibodies

All the antibodies listed below were used in accordance to the manufacturer's instructions: ERα (Santa Cruz, sc-787), ERβ (Abcam, #455), Cyclin B1 (Santa Cruz, sc-7393), p21 (Santa Cruz Biotechnology, INC sc-817), Caspase-3 (Cell Signaling, #9665), PARP (Cell Signaling, #9532), Phalloidin (Alexa Fluor 488, #A12379), Wee1 (Santa Cruz, #5285), p-H2AX (Cell Signaling, #9718), TWIST (Abcam, clone 2C1a #ab50887), SLUG (Santa Cruz, sc-166476), E-cadherin (clone 36 BD #610181), E-cadherin (HECD-1, Abcam, ab1416), β-actin (Sigma-Aldrich, Clone AC-15#A5441) and Tubulin (Sigma-Aldrich clone B-5-1-2 T5168). As secondary antibodies, we used goat anti-mouse IgG (H+L) (#115-035-166, Jackson ImmunoResearch) and goat anti-rabbit IgG (H+L) (#65-6120, Invitrogen) for Western Blot analysis, anti-mouse AF488 (#A11029) for IF analysis and AF647 (#A21235) for cytometry (Alexa Fluor, Molecular Probes Eugene, OR, USA).

### Bioinformatics analysis

The Illumina HiSeq RNA-seq dataset of 471 patients with Skin Cutaneous Melanoma (SKCM) was downloaded from the GDC Data Portal 26 by the TCGAbiolinks R package (ver 2.18.0) 27. According to the subtype mutation, we extracted from the SKCM dataset the BRAFmut (patients with BRAF V600E mutation) and NRASmut (patients with NRAS Q61R mutation) subsets. Data preprocessing, normalization and quantile filtering was performed on each subset. By the TCGAbiolinks command embedding the edgeR R package (ver 3.32.1) 28, we performed a differential expression analysis between Metastatic and Primary tumors in all subsets to investigate ERα and ERβ gene expression across conditions, applying a false discovery rate (FDR) threshold < 0.15. The ratio between ERα and ERβ expression was also calculated in all the subgroups. Additional comparisons of ERα and ERβ gene expression as well as their ratio among patients with primary or metastatic tumor were performed by Generalized Liner Models analysis in R setting significance p < 0.05.

### Statistical Analysis

Unless indicated otherwise, all data are presented as mean ± standard deviation (SD) and results are representative of at least three independent experiments. Statistical analysis was performed using t-test, with p<0.05 deemed statistically significant.

## Results

### ESR1 and ESR2 expression in SKCM patients

**Figure 1:**
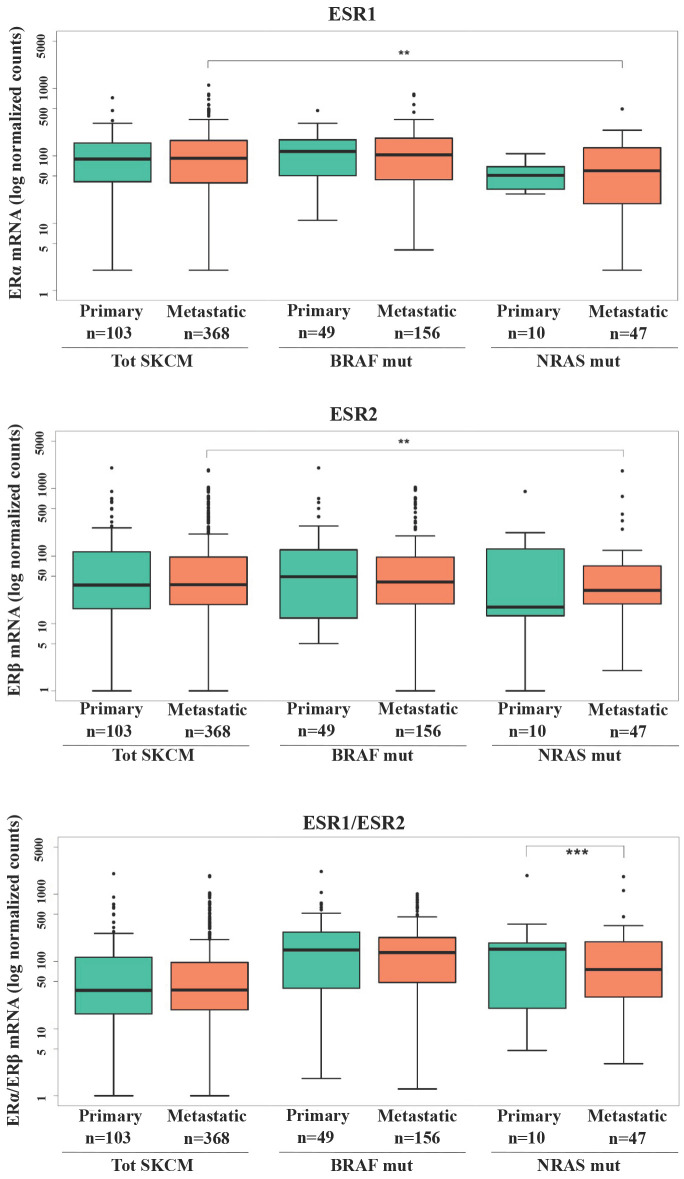
** ERα and ERβ expression in SKCM patients***.* RNA-seq counts in logarithmic scale in total SKCM, BRAF and NRAS mutated patients with primary or metastatic tumors for ERα gene expression (ESR1), ERβ gene expression (ESR2) and ratio between ERα and ERβ gene expressions. Data are reported as interquartile (IQ) boxplots with inbox lines indicating median values and whiskers ± 1.5 IQ range. Asterisks indicate the level of significance: **p < 0.01; ***p<0.001.

Several efforts have been done in evaluating the expression levels of ERs in relation to the stage of melanoma progression with minor attention regarding the mutational state. Investigation of ERα and ERβ gene expression (ESR1 and ESR2, respectively) in SKCM patients from TCGA database (Fig. 1) revealed that no significant difference in ER1/2 gene levels was recorded between primary and metastatic patients in total SKCM, as well as BRAF or NRAS mutated subgroups. However, subjects with metastatic NRAS mutated tumors had significantly lower ERα (p<0.01) and ERβ (p<0.01) expressions compared to the total population of subjects with metastatic melanoma (Fig. 1). Regarding ERα, a decrease with borderline significance (p = 0.054) was recorded between patients with NRAS and BRAF primary tumors (Fig. 1). Since the balance between the two ERs expression might be more representative in defining estrogenic tissue-specific signaling, we analyzed the ERα/ERβ ratio between primary and metastatic melanoma both in the whole melanoma population and in the mutated subgroups. The significantly lower value observed in NRAS metastatic than in primary samples (p<0.001) (Fig. 1) was suggestive of a more pronounced down-regulation of ERα compared to ERβ in the advanced tumor stage.

### ERα and ERβ expression in melanoma cell lines

**Figure 2:**
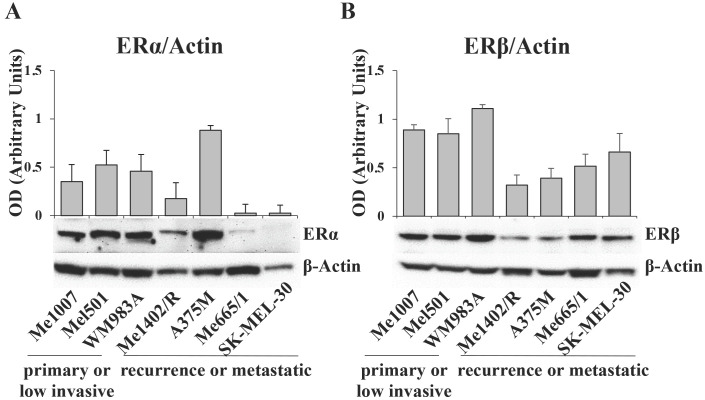
** ERα and ERβ expression levels in different tumor staged melanoma cell lines.** Western blot analysis of (**A**) ERα and (**B**) ERβ in primary (Me1007, Mel501, WM983A), recurrence (Me1402/R) and metastatic (A375M, Me665/1, SK-MEL-30) melanoma cell lines, and corresponding relative densitometric quantification. β-Actin was utilized as internal loading control. Data are expressed as the mean + SD of three independent analyses.

The data obtained from TCGA analysis on melanoma patients led us to investigate ERα and ERβ expression levels in a panel of human melanoma cell lines stabilized from tumors at different stages of progression and genetic background (Table S1). By western blot assay, we detected high levels of ERα in the primary wild type melanoma lines Me1007 and Mel501 and in the BRAF mutated WM983A, it was weakly visible in Me1402/R, a mutated BRAF recurrence of primary melanoma, and barely detectable in NRAS-mutated metastatic Me665/1 and SK-Mel 30 cell lines (Fig. 2A). Interestingly, the highly invasive BRAF-mutated A375M showed a very high level of this protein, in agreement with ERα tumorigenic potential. Regarding ERβ, we observed comparable levels in primary melanomas and in NRAS mutated metastatic cell lines, and lower, but well detectable levels in the BRAF mutated primary Me1402/R and metastatic A375M cell lines (Fig. 2B). Based on these results, we selected three melanoma cell lines for further studies, as representative of three different pathological conditions. We chose A375M and Me665/1, different in terms of genetic mutations but both extremely aggressive, as metastatic cell lines, together with Me1402/R melanoma cells, characterized by low expression of the oncomiR-221 and -222 and capability to melanin pigment synthesis 29, as a good model of primary melanoma capable to progress to a metastatic aggressive form.

### Effects of Estrogen receptor β selective agonist (LY500307) on melanoma cell lines proliferation and viability

In order to investigate the potential ERβ antitumor role in melanoma upon receptor specific hormonal activation, we evaluated the effects of the specific ERβ agonist LY500307 on cell proliferation/viability of Me1402/R, A375M and Me665/1 melanoma cell lines (Fig. S1). Normal human primary fibroblast cell line (HF) was included in the viability assay, to exclude a non-specific toxic effect of LY500307 on cell proliferation. These cell lines were incubated with increasing doses of LY500307 (2µM, 4µM, 8µM) and cellular viability assessed at different time points. An initial reduction of the viability/proliferation index was observed in Me1402/R already after 24 hours of treatment followed by a strong increase in the following 48 and 72 hours. Concerning the metastatic cells, LY500307 treatment significantly reduced cell proliferation of Me665/1 as early as at 24 hours, whereas 48-72 hours of treatment were necessary to detect a significant proliferative rate reduction in A375M. Notably, no viability decrease was observed in normal fibroblasts at all the tested doses and time points of treatment, suggesting LY500307 particular effectiveness on melanoma proliferation and viability, being nearly ineffective on normal cells.

### LY500307 affects melanoma cell cycle

**Figure 3:**
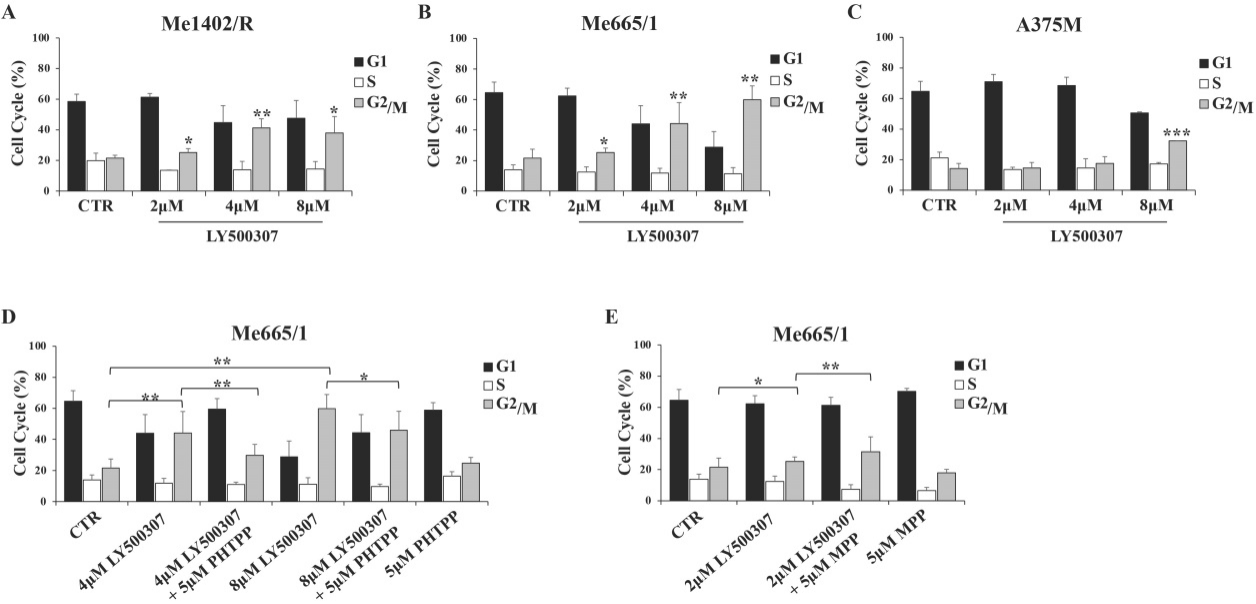
**LY500307 and its combined treatment with ERβ/α antagonists (PHTPP, MPP) on melanoma cell cycle.** Cell cycle analysis of control (CTR) vs cells treated with increased concentrations of LY500307 (2μM, 4μM, 8μM) in (**A**) Me1402/R, (**B**) Me665/1 and (**C**) A375M cell lines for 24 hours. Cell cycle analysis of Me665/1 cells treated with either (**D**) PHTPP (5μM) and LY500307 (4μM or 8μM) or (**E**) MPP (5μM) and LY500307 (2μM) alone or in combination for 24 hours respect to the untreated control (CTR). Data are represented as mean ± SD of three independent experiments. Asterisks indicate the level of significance: * p<0.05; ** p<0.01; *** p<0.001 compared with the corresponding CTR or LY500307 treated cells.

In view of the observed LY500307 effects on melanoma proliferation rate, we analyzed the cell cycle profile of the same cell lines in response to increasing concentration of LY500307 (from 2 to 8 μM). To this purpose, we cultured melanoma cells for three days in presence of 2% FBS DMEM thereafter adding LY500307 at the indicated concentrations. After 24 hours of treatment, we observed by flow cytometry analysis of PI-stained cells, a significant increase of Me1402/R cell percentage at the G2/M boundary phase compared to vehicle-treated controls at all LY500307 doses (Fig. 3A). More significant blockage at G2/M boundary phase was detected in Me665/1, especially at the higher concentrations (Fig. 3B), whereas A375M cell cycle was barely affected only at highest LY500307 dose (Fig. 3C). Similar results were obtained treating Me665/1 in medium with 2% charcoal serum, in order to provide hormone-free cell culture conditions (Fig. S2A). The time course of LY500307 treatment confirmed the greater sensitivity of Me665/1 cells that began to accumulate in G2/M boundary phase already after 5 hours. This effect was detectable to a lesser extent in Me1402/R and not at all in A375M cells (data not shown). Similar results were obtained in the SK-Mel 30, another NRAS-mutated metastatic melanoma cell line (Fig. S3A).

Since LY500307 was described as a potent and selective ERβ agonist 20, to confirm our data we performed some competitive experiments with the highly specific antagonist of ERβ, PHTPP 30 on Me665/1, as best responsive cell line. Me665/1 cells were treated with either LY500307 (4 μM and 8 μM) or PHTPP (5 μM), alone or in combination, for 24 hours. Results indicated that the co-administration of LY500307 and PHTPP significantly reduced the effect obtained on cell cycle with LY500307 alone, confirming its selectivity for the ERβ isoform (Fig. 3D). The PHTPP inhibitory effect was stronger at 4 μM LY500307, possibly because at 8 μM dose the G2/M block was too advanced.

Despite the low ERα level in Me665/1, we wondered if the combined treatment of Me665/1 with LY500307 and the selective ERα antagonist MPP (5 μM) 31 could improve the effect of LY500307 treatment. Interestingly, the effect of the ERβ agonist on cell cycle was improved by blocking ERα, whereas MPP treatment was ineffective when used alone (Fig. 3E).

**Figure 4:**
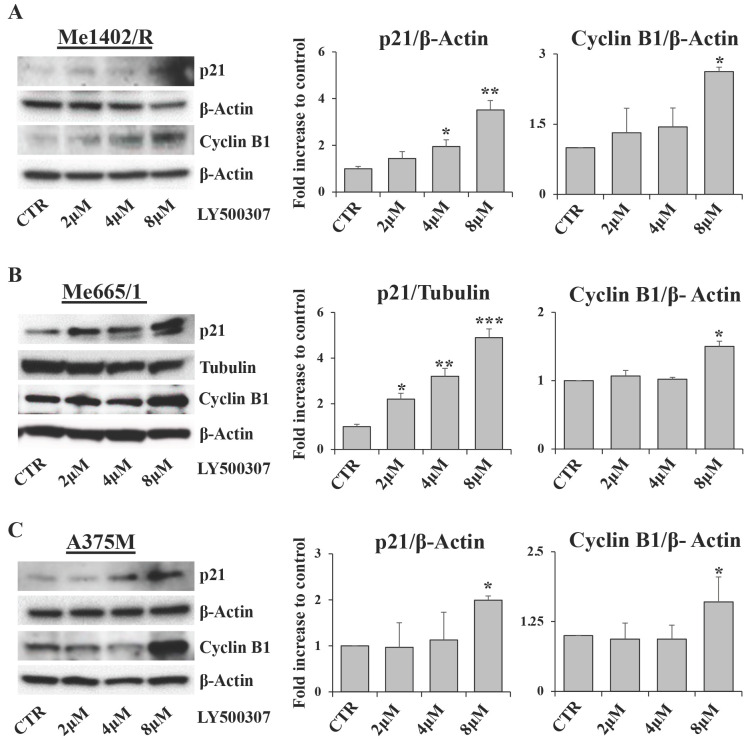
**Evaluation of cell cycle regulators.** Representative Western Blots of p21 and cyclin B1 expression levels in Me1402/R (**A**), Me665/1 (**B**) and A375M (**C**) cells treated with increased concentrations of LY500307 (2μM, 4μM, 8μM) for 24 hours. β-Actin and Tubulin were utilized as internal loading control. Densitometric quantifications shown as fold increase are represented as mean + SD of three independent experiments. Asterisks indicate the level of significance: * p<0.05; ** p<0.01; *** p<0.001 compared with the control cells (CTR).

Cell cycle arrest is characterized by the modulation of several cell cycle regulators 32. Among them, relevant roles are played by p21, a well-known inhibitor of cyclin dependent kinases, and Cyclin B1, whose expression and cellular localization are finely regulated during the cell cycle phases 33. Western blot analysis of p21 expression showed a direct correlation between p21 up regulation and drug amount in all melanoma cell lines treated with LY500307. This modulation was more evident in Me665/1 (Fig. 4B) and SK-Mel 30 cell lines (Fig. S3B) characterized by NRAS mutation, and less clear in Me1402/R (Fig. 4A) and A375M (Fig. 4C) BRAF-mutated cells. These results are consistent with the variability of responses to LY500307 treatment in terms of cell cycle progression in the selected cell lines. Conversely, Cyclin B1 expression remained constant in all the analyzed melanoma cell lines except at 8 μM dose, where it suddenly increased (Fig. 4A, B, C). Regarding Cyclin B1, similar results were obtained in SK-Mel 30 cell line (Fig. S3B) and in Me665/1 treated in culture medium supplemented with charcoal (Fig. S2B). Since cyclin B1 levels oscillate over the course of the cell cycle, being degraded on metaphase-anaphase transition 34, its constant expression observed at 2 and 4 μM and its up-modulation at 8 μM of LY500307 is in line with the G2/M phase arrest.

### LY500307 drives melanoma cells towards mitotic catastrophe events

**Figure 5:**
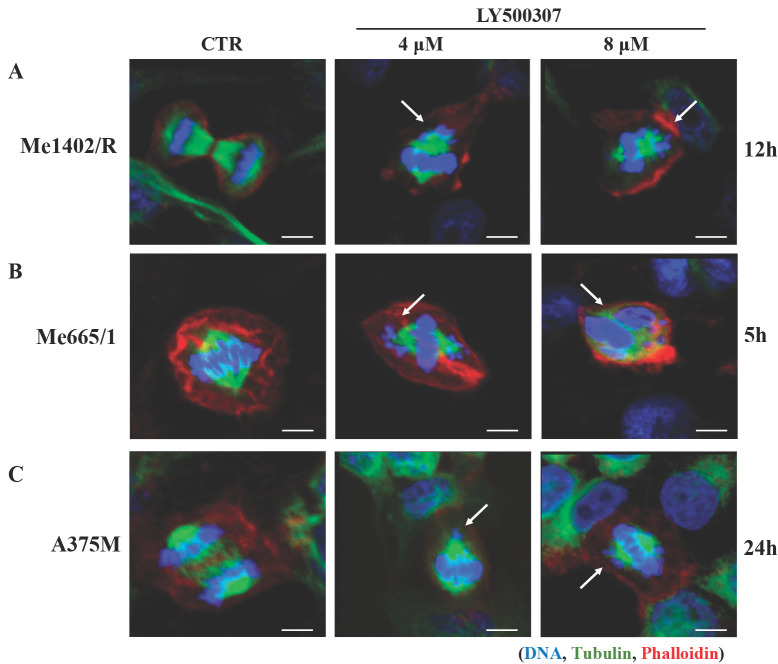
** LY500307 treatment induces alteration in mitotic figures.** Confocal microscopy visualization of cellular and nuclear morphology of (**A**) Me1402/R, (**B**) Me665/1 and (**C**) A375M melanoma cell lines after 12, 5 and 24 hours of LY500307 treatment, respectively. Cells were stained with Phalloidin (Alexa Fluor 647-red) and α-tubulin (Alexa Fluor488-green) for visualization of actin and tubulin filaments, respectively. Nuclei were counter-stained with Hoechst 33342 (blue). Scale bar: 10μM

The observed cell cycle arrest at G2/M boundary upon LY500307 treatment led us to investigate the nuclear morphology of melanoma cell lines with particular attention to the presence of macroscopic anomalies in the mitotic figures. We performed immunofluorescence analyses focusing on DNA and mitotic spindle structure, based on Hoechst and tubulin staining, respectively. Confocal analysis showed that LY500307 treatment determined anomalous mitosis, starting from misaligned metaphasic plaques, up to the formation of small nuclear bodies containing DNA fragments, similar to a mitotic catastrophe event. In agreement with the effect of LY500307 on the cell cycle, we observed abnormal mitosis both in Me1402/R (Fig. 5A) and in Me665/1 (Fig. 5B) after 12 and 5 hours of treatment, respectively. As expected, only after 24 hours of treatment with the highest dose of LY500307, few defective mitoses were detectable in the less responsive A375M cells (Fig. 5C).

**Figure 6:**
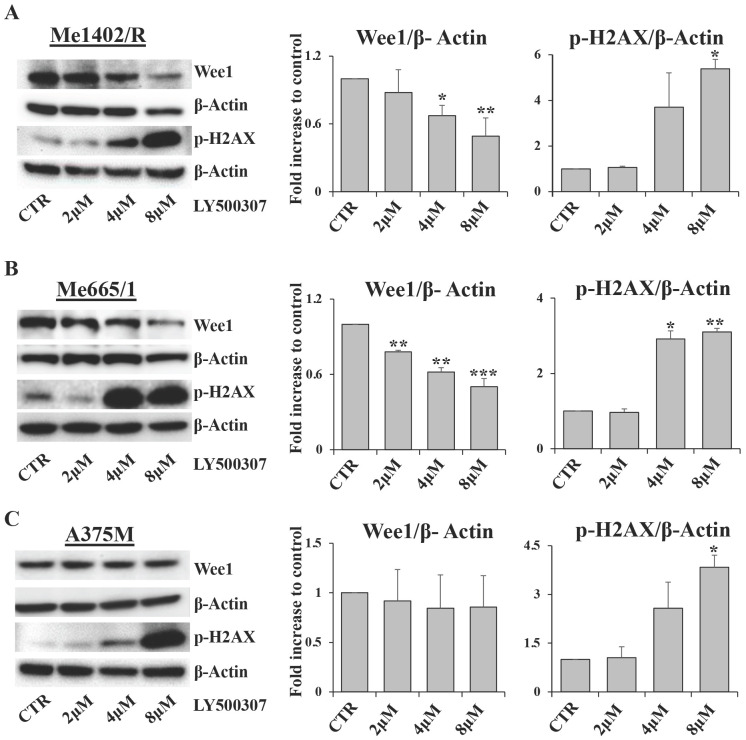
** LY500307 treatment modulates the expression levels of DNA damage related proteins.** Representative Western Blot analysis of Wee1 and p-H2AX expression levels after 24 hours of treatment with increased concentrations of LY500307 (2μM, 4μM, 8μM) in (**A**) Me1402/R, (**B**) Me665/1 and (**C**) A375M cells. β-Actin was utilized as internal loading control. Densitometric quantifications, shown as fold increase, are represented as mean + SD of three independent experiments. Asterisks indicate the level of significance: * p<0.05, ** p<0.01, *** p<0.001 compared with the control cells (CTR).

As cell cycle arrest consists of a series of events often activated by cells in response to DNA damage, we focused our attention on Wee1-like protein kinase (Wee1) and phospho-H2A histone family member X (p-H2AX), two key factors involved in this process. Wee1 is a mitotic inhibitor 35 and H2AX is a histone variant undergoing a rapid phosphorylation in case of DNA injury 36. Western blot analysis showed a significant inverse correlation between Wee1 expression and LY500307 treatment in both Me1402/R and Me665/1, but not in A375M (Fig. 6A, B, C). On the other hand, we found a sharp increase of p-H2AX starting from 4μM of LY500307 in Me665/1 (Fig. 6B) and, to a lesser extent, in Me1402/R and A375M cell lines (Fig. 6A, C).

The opposite expression profiles of Wee1 and p-H2AX suggest that LY500307 might induce the defective cells to bypass DNA damage repair processes and to enter prematurely in mitosis, resulting in improper chromosome segregation eventually leading to mitotic catastrophe and apoptosis.

### LY500307 induces apoptosis in melanoma cells

**Figure 7:**
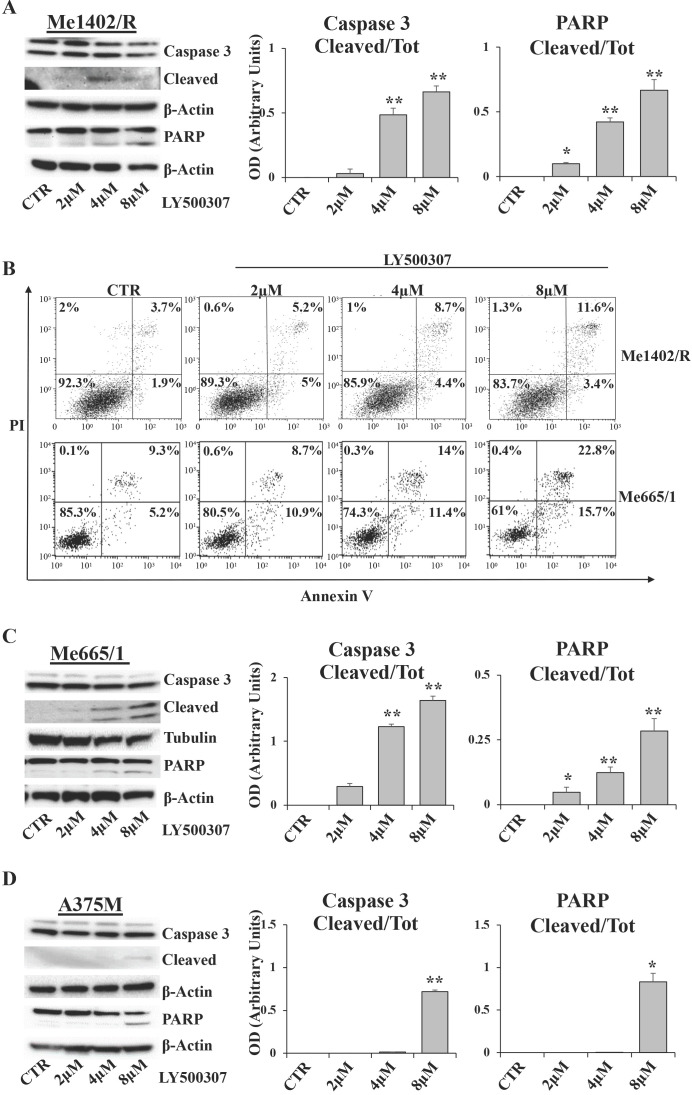
** LY500307 induces apoptosis in Me1402R cell line.** Representative Western Blots illustrate expression levels of Caspase 3, PARP and their cleaved forms in Me1402/R (**A**), Me665/1 (**C**) and A375M (**D**) melanoma cells exposed to increased concentrations of LY500307 (2μM, 4μM, 8μM) for 24 hours. Densitometric quantifications of Cleaved/Total Caspase-3 and PARP ratios are represented as mean + SD of three independent experiments. Quantification of apoptotic cell fractions of Me1402R and Me665/1 (**B**) cells after 24 hours of treatment with either vehicle or LY500307 (2μM, 4μM, 8μM), by Annexin V-FITC and Propidium Iodide (PI) staining and FACS analysis. A representative experiment out of three is shown. β-Actin and Tubulin were utilized as internal loading control. Data are represented as mean + SD of at least three independent experiments. Asterisks indicate the level of significance: * p<0.05, ** p<0.01, *** p<0.001 compared with the control cells (CTR).

In order to evaluate whether melanoma cells blocked in G2/M undergo apoptosis, we analyzed by western blot some specific apoptotic markers, such as caspase 3 and Poly (ADP-Ribose) Polymerase (PARP), and, in parallel, the amount of Annexin V/PI positive cells by Flow Cytometry (FCM). After 24 hours of LY500307 treatment, Me1402/R showed a dose dependent cleavage of both caspase 3 and PARP, statistically significant at 4μM and 8μM of LY500307 (Fig. 7A). Accordingly, FCM analysis showed a progressive increase of late-apoptotic cells (Annexin V/PI-double-positive cells), directly proportional to the treatment dose (Fig. 7B).

Me665/1 cells showed activation of both caspase 3 and cleaved PARP (Fig. 7C). FCM analysis of PI/Annexin V, showed a sharp increase of both early- and late-apoptotic cells (Annexin V^+^/PI^-^ and Annexin V^+^/PI^+^ -positive cells) proportional to LY500307 concentration (Fig. 7B).

Conversely and according to cell cycle analysis, A375M cells revealed a slight activation of PARP and caspase 3 only at 8μM of LY500307 (Fig. 7D) and no increase of PI/Annexin V positive cells (data not shown).

### LY500307 counteracts melanoma cell motility and modulates the expression of epithelial-mesenchymal transition transcription factors (EMT-TFs)

**Figure 8:**
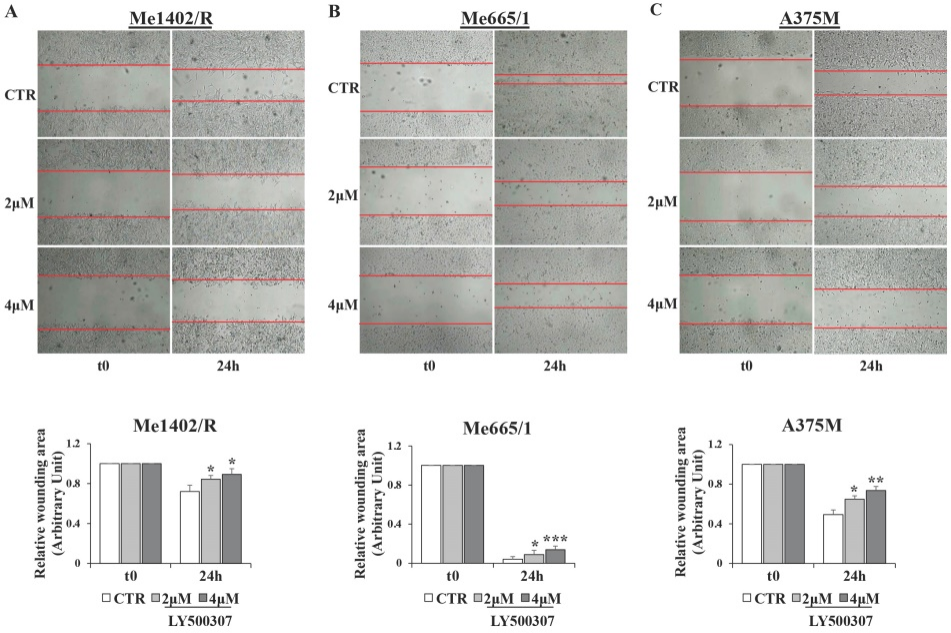
** Analysis of cell migration by scratch assay in melanoma cell lines after LY500307 treatment**. Representative time-lapse microscopy images (upper panel) of wound closure of (**A**) Me1402R (**B**) Me665/1 and A375M (**C**) cells, untreated and treated with LY500307 (2μM and 4μM) for 24 hours. Bar graph of corresponding relative wounding area (lower panel). Results represent the mean + SD of at least four measurements of each wounded area, obtained in three independent experiments. Asterisks indicate the level of significance: * p<0.05, ** p<0.01, *** p<0.001 compared with the control cells (CTR).

**Figure 9:**
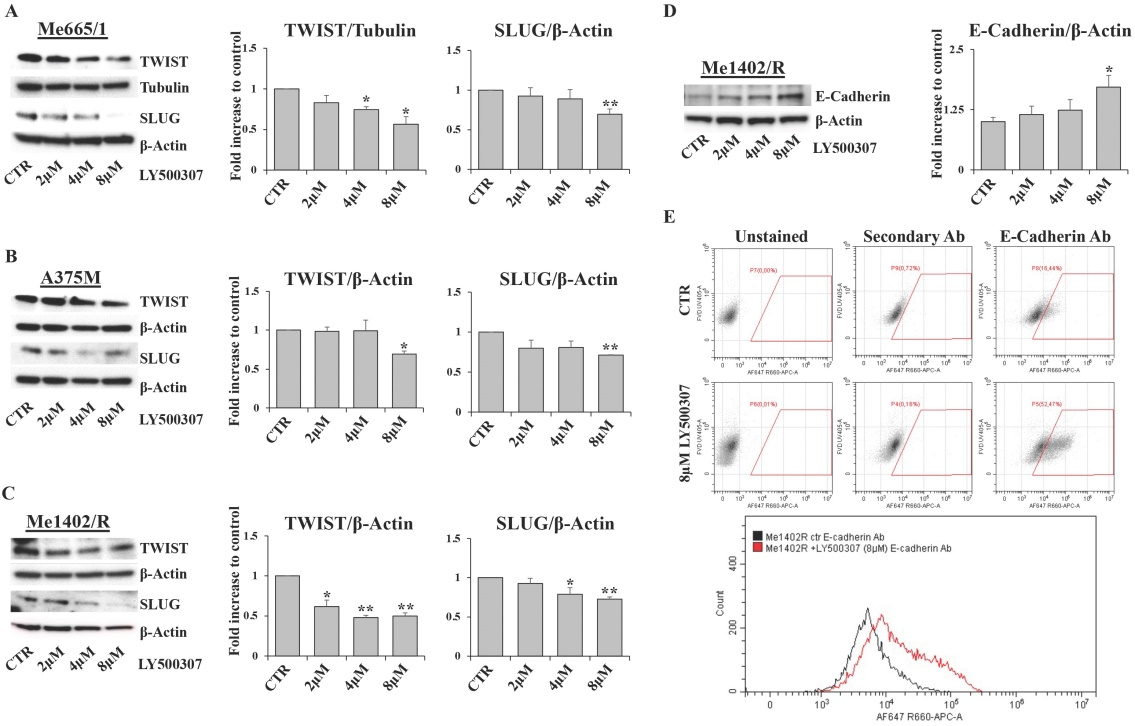
**Effect of LY500307 treatment on EMT-TFs.** Representative WB analysis of TWIST and SLUG in Me665/1 (**A**), A375M (**B**) and Me1402/R (**C**) after 24h of treatment with increasing doses of LY500307 (2μM, 4μM, 8μM). E-cadherin expression analysis by WB (**D**) and flow cytometry (**E**) (CTR vs 8μM LY500307) in Me1402/R cells treated for 24 hours with LY500307. A representative FCM experiment out of three is shown. β-Actin or Tubulin were utilized as internal loading control. Densitometric quantifications shown as fold increase are represented as mean + SD of three independent experiments. Asterisks indicate the level of significance: * p<0.05, ** p<0.01 compared with the control cells (CTR).

Migration and invasion capabilities of tumor cells play an important role in spreading and metastasization 37 and ERβ activation has often been involved in epithelial versus mesenchymal transition inhibition, as in breast and prostate cancers 38, 39. Therefore, by *in vitro* scratch assays performed in low serum condition, we evaluated the effect of LY500307 on the migration ability of melanoma cell lines (Fig. 8A, B, C). Interestingly, we observed a significant dose-dependent reduction of cell migration with respect to the corresponding untreated cells, irrespective of the closure or not of wound area in the controls. Particularly, for the less aggressive Me1402/R cells, it is conceivable that low serum culture condition per se influenced the rate of cell migration (Fig. 8A). Since EMT underlies the increased aggressiveness of advanced tumors, that lose the epithelial characteristics acquiring the mesenchymal ones, and based on the reduced dissemination of LY500307-treated cells, we investigated whether LY500307 could modulate some key transcription factors (i.e. SLUG and TWIST) as well as the adhesion molecule E-cadherin, favoring a mesenchymal-epithelial transition (MET) 40. Indeed, although at different levels, western blot analysis demonstrated a general dose-dependent decrease of SLUG and TWIST expression in melanoma cells (Fig. 9A, B, C).

Interestingly, in Me1402/R the decreased expression of these EMT-TFs was associated with a gradual increase of E-cadherin expression, which became significant at the highest concentration of LY500307 (Fig. 9D). It is important to evidence that this increase was associated with exposure of E-cadherin on cell membrane surface, as demonstrated by flow cytometric analysis of viable cells stained with antibody recognizing the extracellular domain of E-cadherin (Fig. 9E). This last result is suggestive of epithelial-mesenchymal reversion onset in Me1402/R cells, an event conceivable for a local relapse-derived melanoma cell line with most of the characteristics of the primary tumor.

## Discussion

Epidemiological data show a significant gender difference in melanoma incidence, partly based on the pivotal role played by sex hormones 18. In this respect, ERβ displays a suppressive activity 41 and low expression of its mRNA correlates with poorer relapse-free survival of melanoma patients 42. Regarding ERα, an interesting association between its gene polymorphisms and some clinical parameters has been shown 43. To our knowledge, no information has been reported so far on a possible correlation between ER1/2 expression and mutational status of melanoma. In this regard, our analysis, conducted on the TCGA database, did not show a significant difference in ER expression associated with melanoma progression. Nevertheless, significantly lower levels of both ERα and ERβ were found in the subgroup of metastatic NRAS mutated samples compared to the total metastatic population. Of note, the lower ERα/β ratio observed in metastatic versus primary NRAS mutated melanomas suggests a greater decrease of ERα than ERβ expression. In line with that, ER protein levels evaluated in different melanoma cell lines, showed the lowest ERα amount in NRAS metastatic cells Me665/1 and SK-Mel 30, whereas ERβ, albeit lower respect to primary tumor, remained well detectable. Conversely to our TCGA analysis, the BRAF-mutated A375M showed an elevated expression of ERα, thus representing a model of metastatic melanoma, opposite to the NRAS ones in our study. As reported before, Zhao and colleagues, showed the capability of the synthetic non-steroidal selective ERβ agonist LY500307 to reduce melanoma lung metastasis in a murine melanoma *in vivo* model, by innate immunity upregulation in the metastatic niche 19. Although of interest, this study was focused on B16F10 mouse metastatic melanoma cell line, wild-type respect to either BRAF or NRAS mutations, and indicated the tumor microenvironment as key mediator of LY500307 effects, without evidence of any cell-dependent pathway activation. Therefore, in order to translate the LY500307 function to human disease, we focused our study on some representative human melanoma cell lines.

Here, we demonstrated the ability of this compound to affect melanoma cell proliferation with minimal toxicity on normal cells (Fig. S1), inducing cell cycle arrest in G2/M phase in agreement with the absolute amount of ERβ or the relative expression of ERα and ERβ in each melanoma cell line (Fig. 3A, S2). Interestingly, the ERβ antagonist PHTPP (Fig. 3D) was able to attenuate LY500307 effect and, possibly more important, the ERα antagonist MPP increased the cell sensitivity to a lower dose of LY500307 (Fig. 3E). Suppression of proliferation through blockade of cell cycle progression has also been observed in other two sex hormone-related cancers, as glioblastoma 20 and triple negative breast cancer (TNBC) 21.

Interestingly, this G2/M cell cycle block was associated with reduced Wee1 kinase expression and induction of histone H2AX phosphorylation (Fig. 6). Hence, we can assume that this blockage is a consequence of deficiency in some cell cycle checkpoints leading cells to enter mitosis before genomic instability resolution and in turn undergoing mitotic catastrophe. Interestingly and differently to what observed in the B16-based mice model 19, LY500307 was able to induce a cell-autonomous apoptotic program in these *in vitro* treated human melanoma cell lines. Therefore, LY500307 appears to exert a double activity against cancer, decreasing tumor growth and increasing the availability of tumor specific antigens to be recognized as non-self by the immune system, as shown by reduced proliferation and apoptosis onset. To note, a recent study in murine Triple-Negative Breast Cancer (TNBC) and colorectal cancer cells demonstrated that combined treatment of ERβ agonist and PD-1 antibody, reduced Myeloid-Derived Suppressor Cells (MDSC) tumor infiltration and enhanced its response to ICB therapy 44.

According to the supposed ERβ antimetastatic activity, LY500307 resulted able also to reduce cell migration (Fig. 8) and down-modulate some of the transcription factors involved in the epithelial-mesenchymal process (Fig. 9). Interestingly, in the less aggressive Me1402/R cells, E-cadherin was increased and correctly localized on the membrane surface, suggestive of a more complete mesenchymal-epithelial reversion (Fig. 9D, E), easier to obtain *in vitro* in this melanoma cell line, with still some molecular traits typical of primary stage. It is worth noting that ERα did not counteract ERβ functional activity on the invasion capacity suggesting that both cell migration and MET could be modulated by ERβ agonist via molecular pathways not influenced by ERα signaling. Accordingly, reduced cell migration associated with E-cadherin upregulation by ERβ expression and further increased by LY500307 treatment was recently demonstrated in TNBC 45.

Overall, our work demonstrated the oncosuppressive effect of ERβ activation on some representative human melanoma cell lines, characterized by different levels of ERs expression, genetic background and tumor progression level.

Further *in vivo* studies will shed light on the possible role of ERβ in counteracting melanoma metastasization, considering the possible influences of the tumor microenvironment besides the direct actions of LY500307 here reported on melanoma cells. Indeed, although impressive therapeutic results have been achieved for the treatment of melanoma over the past decade, effective clinical results are still lacking for NRAS-positive melanoma patients. This is partly due to the low number of NRAS positive patients, which also limited ER expression studies, especially in view of a sex- and age-stratified investigation. As suggested by data obtained with the ERβ agonist LY500307, we could consider the option of ERβ as a candidate for alternative and combined therapeutic strategies. A definite comprehension of the molecular pathways underlying the ERβ effectiveness in mutated NRAS cells would dissect the direct and indirect mechanisms, exploiting the ERβ antitumor potential possibly associated with the different mutations underlying melanoma.

## Conclusions

Our results demonstrate that the selective ERβ agonist LY500307 differently affects melanoma cell proliferation, related to stage, genetic background and ERs expression. These data confirm the ERβ ability to counteract melanoma onset and progression, suggesting the importance of determining both the patient's genetic profile and ERs expression in tumor tissues for selecting the most effective and personalized therapeutic approach.

## Supplementary Material

Supplementary figures.

## Abbreviations

BRAF: B-Raf Proto-Oncogene
CDK4/6: Cyclin Dependente Kinase 4/6
EMT: Epithelial-Mesenchymal Transition
EMT-TFs: Epithelial-Mesenchymal Transition Transcription factors
ERs: Estrogen Receptors
FCM: Flow cytometry
HF: Human Fibroblast
ICIs: Immune Checkpoint Inhibitors
MAPK: Mitogen-Activated Protein Kinase
MDSC: Myeloid-Derived Suppressor Cells
MEK: Mitogen-Activated Protein Kinase
MPP: Methyl-Piperidino-Pyrazole
NRAS: NRAS Proto-Oncogene
p-H2AX: phospo-H2A histone family member X
PARP: Poly (ADP-Ribose) Polymerase
PHTPP: Pyrazolo[1,5-α] pyrimidines
PI3K: Phosphoinositide 3-Kinase
SKCM: Skin cutaneous melanoma
TCGA: The cancer genome atlas
TNBC: Triple-Negative Breast Cancer
Wee1: Wee1-Like Protein Kinase
