# Supplementary material for: Different Susceptibilities of Human Melanoma Cell Lines to G2/M Blockage and Cell Death Activation in Response to the Estrogen Receptor β agonist LY500307
Source: J Cancer. 2022 Mar 6;13(5):1573–87. doi: 10.7150/jca.65425 (PMC8965114; doi:10.7150/jca.65425)
Supplement: Supplementary file 1 — Supplementary figures. [file jcav13p1573s1.pdf]

Different Susceptibilities of Human Melanoma Cell Lines to G2/M Blockage and Cell Death Activation in Response to the Estrogen Receptor β agonist LY500307.

Giada Pontecorvi[1], Maria Bellenghi[1], Sabrina Tait[1], Valentina Tirelli[2], Paola Matarrese[1], Gianfranco Mattia[1*], Alessandra Carè[1*#] and Rossella Puglisi[1#]

[1] Center for Gender-Specific Medicine, Istituto Superiore di Sanità, Rome, Italy

[2] Core facilities, Istituto Superiore di Sanità, Rome, Italy

*Correspondence to: A.Carè and G.Mattia, Istituto Superiore di Sanità, Viale Regina Elena 299, 00161 Rome, Italy. E-mail: alessandra.care@iss.it and gianfranco.mattia@iss.it

[#]Equal senior authorship

Supplementary file Legends

Fig. S1 LY500307 effects on tumor-cell proliferation/viability. Melanoma cell lines (Me1402/R, Me665/1 and A375M), and normal human fibroblasts (HFs) were treated with increasing concentrations of LY500307 (2μM, 4μM, 8μM) at different time points and cell viability measured using an XTT assay in comparison with untreated controls (CTR). Data are represented as mean $\pm$ SD of at least three independent experiments. Asterisks indicate the level of significance: * $p<0.05$, ** $p<0.01$, *** $p<0.001$. Western blotting insert shows ER expression in HFs, with β–actin as loading control.

Fig. S2 Effect of ERβ selective agonist LY500307 treatment on melanoma cell cycle. (A) Cell cycle analysis of Me665/1 cultured in 2% charcoal and treated or not (CTR) with increased concentrations of LY500307 (2μM, 4μM, 8μM) for 24 hours. **(B)** Representative Western blot analysis of Cyclin B1 and Wee1 from total proteins extracted from the same experiment. β-Actin was utilized as internal loading control. Asterisks indicate the level of significance: ** $p<0.01$.

Fig. S3 Effect of ERβ selective agonist LY500307 treatment on NRAS metastatic melanoma SK-MEL-30 cell cycle. **(A)** Cell cycle analysis of SK-MEL-30 cultured treated or not (CTR) with LY500307 (4μM, 8μM) for 24 hours. **(B)** Representative Western blot analysis of p21 and Cyclin B1 from total proteins extracted from the same experiment. β-Actin was utilized as internal loading control. Asterisks indicate the level of significance: ** $p<0.01$, *** $p<0.001$.

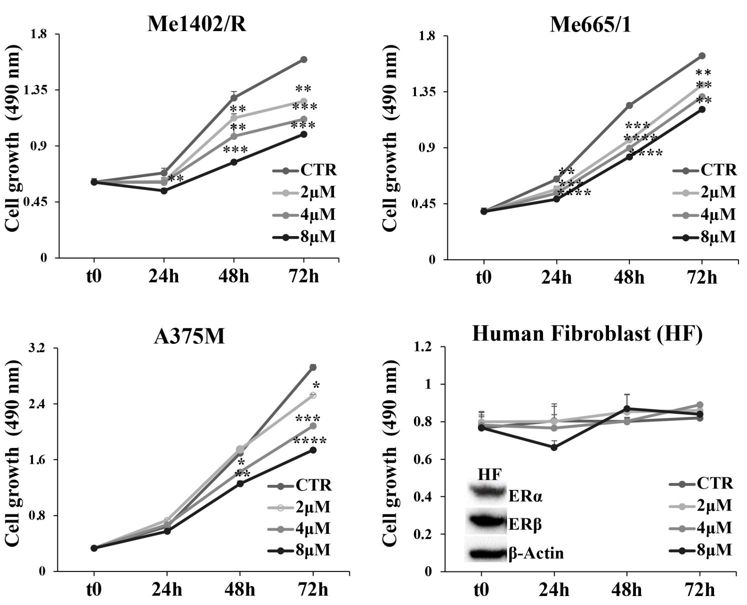

Me1402/R
Me665/1
A375M
Human Fibroblast (HF)
Cell growth (490 nm)
t0
24h
48h
72h
CTR
2μM
4μM
8μM
HF
ERα
ERβ
β-Actin

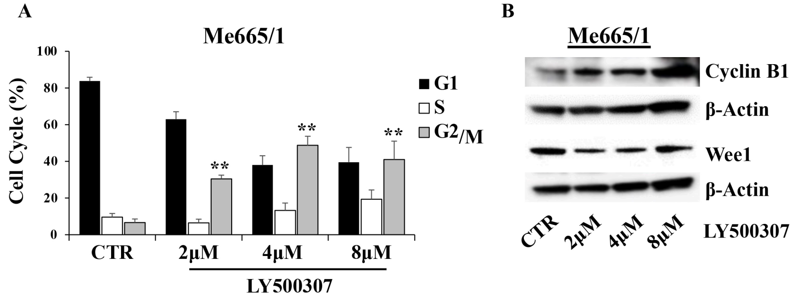

A
Me665/1
Cell Cycle (%)
100
80
60
40
20
0
G1
S
G2/M
**
**
**
CTR
2μM
4μM
8μM
LY500307
B
Me665/1
Cyclin B1
β-Actin
Wee1
β-Actin
CTR
2μM
4μM
8μM
LY500307

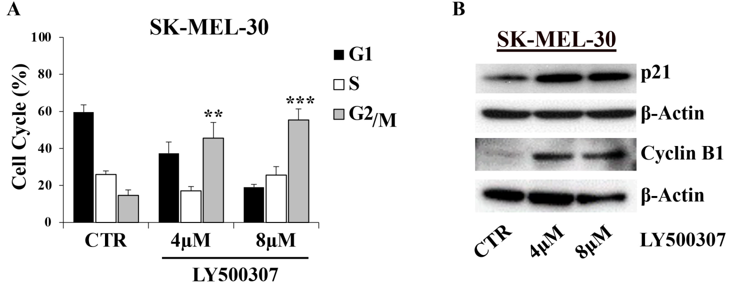
A
SK-MEL-30
Cell Cycle (%)
100
80
60
40
20
0
G1
S
G2/M
**
***
CTR
4μM
8μM
LY500307
B
SK-MEL-30
p21
β-Actin
Cyclin B1
β-Actin
CTR
4μM
8μM
LY500307

Table S1. Melanoma cell lines analyzed in the current study

Cell line	Mutation	Histological features	References
**Me1007**	WT	Primary tumor VGP*	[1, 2, 3]
**Mel501**	WT	Primary tumor VGP*	[4]
**WM983A**	BRAF V600E	Primary tumor VGP*	[4, 5]
**Me1402/R**	BRAF V600E	Recurrence of primary tumor	[1, 2]
**A375M**	BRAF V600E	Metastatic melanoma	[6, 7]
**Me665/1**	NRAS Q61R	Metastatic melanoma	[2, 8]
**SK-MEL-30**	NRAS Q61K	Metastatic melanoma	[9]

*VGP: vertical growth phase

1. Daniotti M, Oggionni M, Ranzani T, Vallacchi V, Campi V, Di Stasi D, et al. BRAF alterations are associated with complex mutational profiles in malignant melanoma. Oncogene 2004;23:5968–77.
2. Colombo MP, Maccalli C, Mattei S, Melani C, Radrizzani M, Parmiani G. Expression of cytokine genes, including IL-6, in human malignant melanoma cell lines. Melanoma Res. 1992;2:181–9.
3. Anichini A, Mazzocchi A, Fossati G, Parmiani G. Cytotoxic T lymphocyte clones from peripheral blood and from tumor site detect intratumor heterogeneity of melanoma cells. Analysis of specificity and mechanisms of interaction. J Immunol. 1989;142:3692-701.
4. Supino R, Mapelli E, Sanfilippo O, Silvestro L. Biological and enzymatic features of human melanoma clones with different invasive potential. Melanoma Res. 1992;2:377–384.
5. Smalley KS, Lioni M, Dalla Palma M, Xiao M, Desai B, Egyhazi S, et al. Increased cyclin D1 expression can mediate BRAF inhibitor resistance in BRAF V600E–mutated melanomas. Mol Cancer Ther. 2008;7:2876–83.
6. Benjamin CL, Melnikova VO, Ananthaswamy HN. Models and mechanisms in malignant melanoma. Mol Carcinog. 2007;46:671–8.
7. Kozlowski JM, Hart IR, Fidler IJ, Hanna N. A human melanoma line heterogeneous with respect to metastatic capacity in athymic nude mice. J Natl Cancer Inst. 1984;72:913–917.
8. Sensi M, Nicolini G, Petti C, Bersani I, Lozupone F, Molla A, et al. Mutually exclusive NRASQ61R and BRAFV600E mutations at the single-cell level in the same human melanoma. Oncogene 2006;25: 3357-64.
9. Tsao H, Goel V, Wu H, Yang G, Haluska FG. Genetic interaction between NRAS and BRAF mutations and PTEN/MMAC1 inactivation in melanoma. J Invest Dermatol. 2004;122:337-41.
